# Machine Learning-Based Mortality Prediction Model Using Minimal Features to Assist Decision-Making in End-of-Life Care

**DOI:** 10.7759/cureus.95874

**Published:** 2025-11-01

**Authors:** Tae Hoon Kong, Jae Ha Kim, Mi Sun Kim

**Affiliations:** 1 Department of Otorhinolaryngology - Head and Neck Surgery, Yonsei University Wonju College of Medicine, Wonju, KOR; 2 Department of Medical Informatics and Biostatistics, Yonsei University Wonju College of Medicine, Wonju, KOR; 3 Department of Radiation Oncology, Yonsei University Wonju College of Medicine, Wonju, KOR

**Keywords:** machine learning, mortality, palliative care, prognosis, radiotherapy

## Abstract

Introduction

International guidelines recommend single or hypo-fraction for palliative radiotherapy (PRT). However, due to the inherent difficulty in accurately predicting life expectancy, the customization of end-of-life care for individual patients poses a significant challenge. This study aims to develop a machine learning-based mortality prediction model for PRT tailored to life expectancy.

Methods

A retrospective analysis encompassed 318 patients who expired after receiving PRT for advanced cancer from March 2013 to July 2023. Various model algorithms employing 22 variables were used to predict mortality within 30 days from initiation of PRT: extra trees, random forest, light gradient boosting machine (LightGBM), and extreme gradient boosting (XGBoost). We evaluated each model's performance using accuracy, precision, recall, specificity, and F1 score.

Results

A total of 302 patients were analyzed after excluding those with missing data. The <30-day mortality group had significantly worse Eastern Cooperative Oncology Group (ECOG) status, lower albumin, higher neutrophil‑to‑lymphocyte ratio, and lower lymphocyte count (all p<0.05). No significant multicollinearity was observed. Among all models, LightGBM showed the best performance (accuracy: 0.725, F1-score: 0.720). A minimal variable model (MVM) using the top eight features plus sex achieved comparable performance to the full variable model (FVM), with improved recall and reduced complexity.

Conclusion

We developed a machine learning-based mortality prediction model for tailoring PRT to life expectancy. The model identified clinically meaningful predictors that reflect patient condition and tumor burden. The MVM demonstrated a comparable performance to the FVM, suggesting its potential utility as an interpretable and practical clinical decision support tool for individualized end-of-life care planning.

## Introduction

In palliative care for patients with advanced cancer, the primary goal of treatment is to alleviate symptoms and enhance quality of life. Palliative radiotherapy (PRT) serves as an effective tool for symptom management, with reports indicating that up to 10% of patients in the terminal stage of cancer undergo PRT within the last 30 days of life [1].

The decision-making process for palliative care involves a multitude of factors, including the patient's medical condition, patients and their family's preferences for end-of-life care, and the physician's prognostic assessment. While evidence-based guidelines typically recommend single or hypo-fractionated radiotherapy without chemotherapy, actual clinical practice often diverges from these guidelines [2]. Optimistic misjudgments regarding a patient's life expectancy may impede the accurate establishment of treatment aims, leading to the administration of prolonged courses of treatment or the administration of chemotherapy. Difficulty in predicting life expectancy for terminally ill patients contributes to this challenge, with a significant number of patients passing away shortly after or during PRT [3].

Artificial intelligence (AI) is increasingly being utilized across various domains in the management of cancer patients, including diagnosis, selection of treatment modalities, and prediction of adverse effects [4,5]. Machine learning-based tools trained on vast amounts of data have the potential to assist physicians in making informed decisions, particularly in clinically challenging situations.

This study aims to develop a machine learning-based mortality prediction model utilizing data from patients with advanced cancer referred for PRT, in order to prescribe proper treatments tailored to each patient's prognosis through data-driven prediction.

## Materials and methods

Subjects and data collection

This study was approved by the institutional review board of our institution (IRB approval number CR322042), and the need for patients’ informed consent was waived due to the retrospective nature of this study.

We retrospectively reviewed the institutional registry and daily radiation oncology department lists between March 2013 and July 2023. PRT was performed on 1361 patients. Patients with hematologic malignancies and those without an exact date of death were excluded. A total of 318 patients who expired during/after receiving PRT for advanced cancer were reviewed. After excluding 16 patients with missing data, a total of 302 patients were included in the analysis. We obtained patient demographics, clinical variables, and treatment information, including age, sex, Eastern Cooperative Oncology Group (ECOG) performance status, and blood test results. The clinical variables were collected at the initiation of PRT. If no measurements were available on the initiation date, the closest available values within two-week window were used. Additionally, we evaluated the following clinical features: the number of metastatic organs involved; presence of liver and bone metastases; performance of palliative surgery; number of prior chemotherapy regimens; concurrent chemotherapy during PRT; number of PRT sessions; prescribed PRT dose and fractionation; hospitalization during the PRT period; hospitalization within three months prior to PRT; admission to intensive care units; cancer-related emergency department visits; and whether the primary tumor originated from the breast or prostate.

Outcome assessment

A predictive model was developed using the aforementioned variables as independent predictors, with the primary outcome defined as mortality within 30 days following PRT. Patients were categorized into two groups based on survival status: those who died within 30 days after PRT and those who survived beyond 30 days, and comparative analyses were performed between these groups. During the analysis, cases with missing values in the independent variables were excluded to ensure that only complete data were used in model development.

Statistical analysis

Continuous variables were compared between the two groups using the independent t-test, while categorical variables were analyzed using the chi-square test. Statistical analyses were performed using Python version 3.12.7 (Python Software Foundation, Wilmington, DE, USA). To assess multicollinearity among variables, exploratory data analysis was conducted to examine pairwise correlations; variables with correlation coefficients ≤0.4 were considered not to exhibit multicollinearity.

Among the 302 patients included in the study, 119 (39.4%) died within 30 days following PRT, whereas 183 (60.6%) died thereafter, indicating a moderate class imbalance. However, no re-sampling or weighting adjustment was applied, as the imbalance was not considered substantial enough to affect model stability.

The dataset was randomly divided into training and testing subsets at a ratio of 7:3. Tree-based models were implemented for prediction, with logistic regression serving as the control model. The machine learning algorithms applied included light gradient boosting machine (LightGBM), extreme gradient boosting (XGBoost), random forest, and extra trees. Model performance was evaluated based on accuracy, precision, recall, specificity, and F1-score. Model performance was further validated using 10-fold cross-validation to enhance generalizability and prevent overfitting. Hyperparameter tuning for each machine learning algorithm was performed using Bayesian optimization, which efficiently explores the parameter space to identify the optimal configurations. We applied recursive feature elimination using a tree-based estimator to iteratively remove less informative variables. The process was repeated until a stable set of predictors maximizing model accuracy and interpretability was identified. To determine the optimal number of features, test accuracy was assessed according to the number of top-ranked features. Variable importance was estimated using the split method, which quantifies importance based on the number of times each feature was used to split nodes across all trees in the model.

## Results

The majority were men (70.2%), and the median age was 64 (interquartile range (IQR) 58-73) years (Table 1). The median age of patients who died within 30 days of PRT was 65 (IQR 59-74) years compared to 64 (IQR 58-73) years in those who survived beyond 30 days, with no statistically significant difference between the two groups (p=0.333). There was also no significant difference in sex distribution between the groups (p=0.950). Lung cancer was the most common primary tumor in both groups. The ECOG performance status was significantly higher in the <30-day mortality group compared to the ≥30-day group (p<0.001) [6]. Serum albumin levels were also significantly lower in the <30-day group (3.3±0.6 g/dL) than in the ≥30-day group (3.9±2.2 g/dL; p<0.001). The neutrophil‑to‑lymphocyte ratio (NLR) was significantly elevated in the <30-day group (10.2±8.0) compared to the ≥30-day group (6.4±5.9; p<0.001). Lymphopenia was more frequent in the <30-day group (53.5%) than in the ≥30-day group (42.3%; p=0.066). Liver metastasis, bone metastasis only, and breast/prostate cancer origin showed no difference between the two groups. Hospitalization during PRT and intensive care unit admission were observed more often in the <30-day group (93.1% and 5.9%) than in the ≥30-day group (69.2% and 1.0%), respectively (p<0.001 and p=0.012) (Table 2). Other treatment-related variables showed no significant intergroup differences.

**Table 1:** Clinical characteristics of patients Abbreviations: IQR: interquartile range; SD: standard deviation; ECOG: Eastern Cooperative Oncology Group.

		Total	Death within 30 days	Death after 30 days	p-value	t-value	Chi-square value
		No. (%)	No. (%)	No. (%)			
No. of patients		302	101	201			
Age					0.333	-0.634	
	Median (IQR)	64 (58–73)	65 (59–74)	64 (58–73)			
Sex					0.950		0.004
	Male	213 (70.5)	71 (70.3)	142 (70.6)			
	Female	89 (29.5)	30 (29.7)	59 (29.4)			
Primary tumor					0.747		5.932
	Lung	188 (62.3)	118 (58.7)	70 (69.2)			
	Gastrointestinal	31 (10.3)	24 (11.8)	7 (6.9)			
	Genitourinary	26 (8.6)	19 (9.5)	7 (6.9)			
	Breast	16 (5.3)	11 (5.5)	5 (5.0)			
	Hepatopancreatobiliary	16 (5.3)	13 (6.5)	3 (3.0)			
	Gynecology	9 (3.0)	5 (2.5)	4 (4.0)			
	Head and neck	8 (2.6)	6 (3.0)	2 (2.0)			
	Soft tissue sarcoma	3 (1.0)	2 (1.0)	1 (1.0)			
	Skin	2 (0.6)	1 (0.5)	1 (1.0)			
	Unknown	3 (1.0)	2 (1.0)	1 (1.0)			
ECOG (grade)					< 0.001		37.217
	0-1	76 (24.1)	3 (3.0)	70 (34.8)			
	2-4	239 (75.9)	98 (97.0)	131 (65.2)			
Serum albumin (g/dL, mean±SD)		3.7±1.7 (3.1–4.0)	3.3±0.6 (2.9–3.7)	3.9±2.2 (3.3–4.1)	< 0.001	5.228	
Neutrophil-to-Lymphocyte Ratio (ratio, mean±SD)		7.9±7.0 (3.1–9.7)	10.2±8.0 (4.8–14.0)	6.4±5.9 (2.7–7.5)	< 0.001	-5.048	
Lymphocyte (10^3^/µL, mean±SD)		1152.7±634.9 (670-1510)	1035.8±582.5 (620-1370)	1228.7±657.2 (710-1615)	0.021	2.321	
Lymphopenia					0.066		3.380
	Yes	139 (46.0)	54 (53.5)	85 (42.3)			
	No	163 (54.0)	47 (46.5)	116 (57.7)			
Number of involved organs					0.117	-1.593	
	Median (IQR)	2 (1-3)	2 (1-3)	2 (1-3)			
Liver metastasis					0.095		2.790
	Yes	75 (24.8)	70 (69.3)	157 (78.1)			
	No	227 (75.2)	31 (30.7)	44 (21.9)			
Bone-only metastasis					0.410		1.188
	Yes	43 (14.2)	11 (10.9)	32 (15.9)			
	No	259 (85.8)	90 (89.1)	169 (84.1)			
Breast/prostate cancer origin					0.506		0.905
	Yes	31 (10.3)	8 (7.9)	23 (11.4)			
	No	271 (89.7)	93 (92.1)	178 (88.6)			

**Table 2:** Treatment characteristics of patients

		Total	Death within 30 days	Death after 30 days	p-value	Chi-square value
		No. (%)	No. (%)	No. (%)		
No. of patients		302	101	201		
Admission during radiotherapy					< 0.001	21.810
	Yes	233 (77.2)	94 (93.1)	139 (69.2)		
	No	69 (22.8)	7 (6.9)	62 (30.8)		
Admission within 3 months before radiotherapy					0.295	1.099
	Yes	194 (64.2)	69 (68.3)	125 (62.2)		
	No	108 (35.8)	32 (31.7)	76 (37.8)		
Intensive care unit admission					0.012	6.376
	Yes	8 (2.6)	6 (5.9)	2 (1.0)		
	No	294 (97.4)	95 (94.1)	199 (99.0)		
Emergency room visit					0.125	2.357
	Yes	18 (6.0)	9 (8.9)	9 (4.5)		
	No	284 (94.0)	92 (91.1)	192 (95.5)		
Palliative surgery performed					0.736	0.114
	Yes	20 (6.6)	6 (5.9)	14(7.0)		
	No	282 (93.4)	95 (94.1)	187(93.0)		
Palliative chemotherapy performed					0.117	2.462
	Yes	226 (74.8)	70 (69.3))	156 (77.6)		
	No	76 (25.2)	31 (30.7)	45 (22.4)		
Number of previous chemotherapy regimens					0.887	0.020
	0-1	199 (65.9)	66 (65.3)	133 (66.2)		
	≥2	103 (34.1)	35 (34.7)	68 (33.8)		
Concurrent chemotherapy with radiotherapy					0.094	2.810
	Yes	131 (43.4)	37 (36.6)	94 (46.8)		
	No	171 (56.6)	64 (63.4)	107 (53.2)		
Hypofractionated radiotherapy performed					0.559	0.341
	Yes	40 (13.2)	25 (12.4)	15 (14.9)		
	No	262 (86.8)	176 (87.6)	86 (85.1)		
Radiotherapy prescribed more than 10 fractions					0.075	3.175
	Yes	79 (26.2)	20 (19.8)	59 (29.4)		
	No	223 (73.8)	81 (80.2)	142 (70.6)		
Multisession radiotherapy performed					0.586	0.297
	Yes	102 (33.8)	32 (31.7)	70 (34.8)		
	No	200 (66.2)	69 (68.3)	131 (65.2)		
Number of radiotherapy sites					0.360	0.837
	Single site	268 (88.7)	92 (91.1)	176 (87.6)		
	Multisite	34 (11.3)	9 (8.9)	25 (12.4)		

Correlation analysis revealed that all pairwise correlation coefficients among independent variables were ≤0.31 in absolute value, indicating no significant multicollinearity. Model performance results are summarized in Table 2. Logistic regression yielded an accuracy of 0.659, precision of 0.727, recall of 0.714, specificity of 0.571, and F1-score of 0.642. Among all models tested, LightGBM demonstrated the best overall performance with an accuracy of 0.725, precision of 0.830, recall of 0.696, specificity of 0.771, and F1-score of 0.720. XGBoost achieved a high recall (0.804) but relatively low specificity (0.457), with an accuracy of 0.670 and F1-score of 0.633. Random forest showed an accuracy of 0.703, recall of 0.821, and F1-score of 0.672, while extra trees yielded an accuracy of 0.692, recall of 0.839, and F1-score of 0.652. While each model had unique strengths, LightGBM provided the most balanced and superior performance.

**Figure 1:**
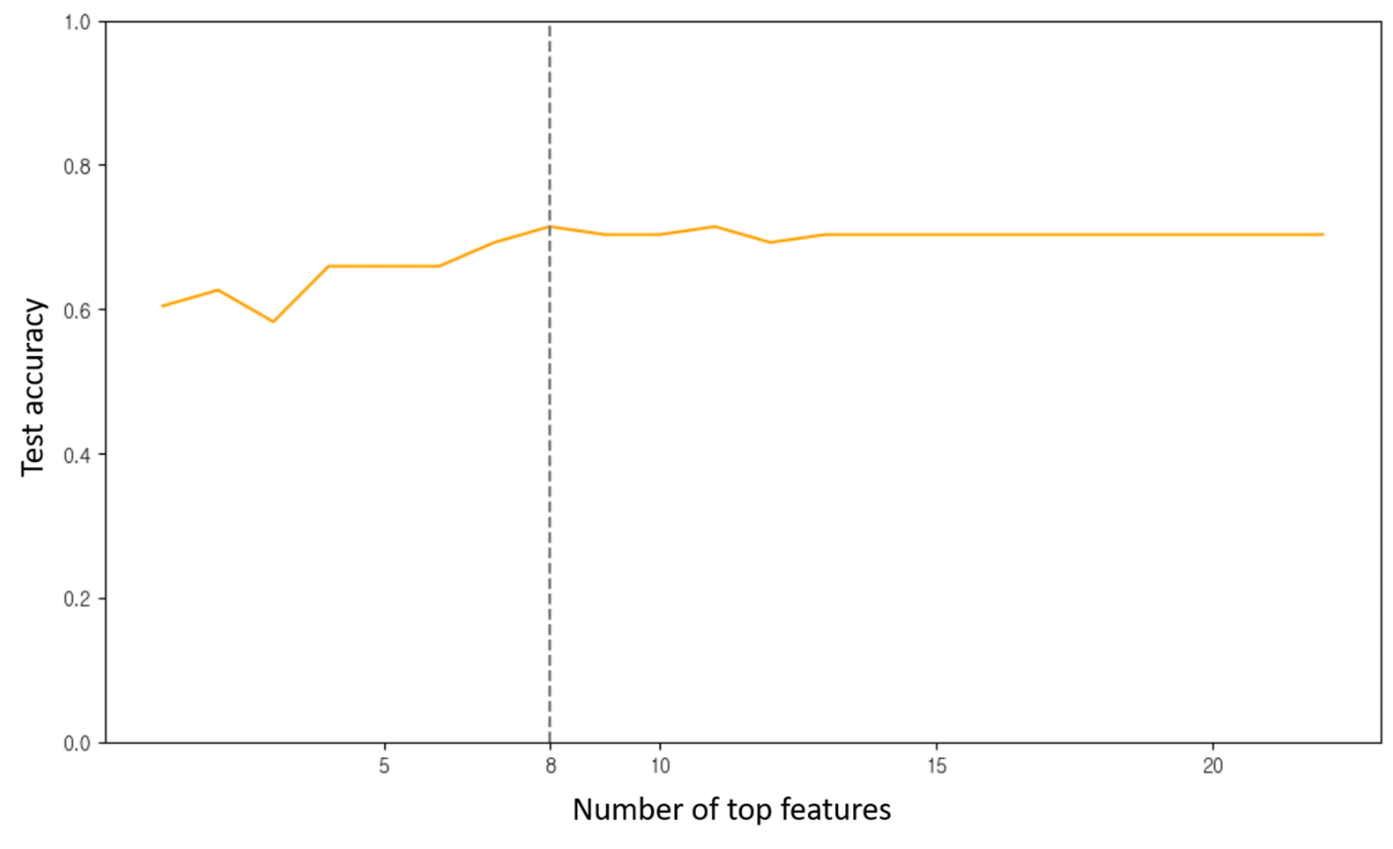
Test accuracy of light gradient boosting machine Test accuracy of the light gradient boosting machine as a function of the number of top features selected based on importance. The highest test accuracy was achieved when the top eight features were used.

**Figure 2:**
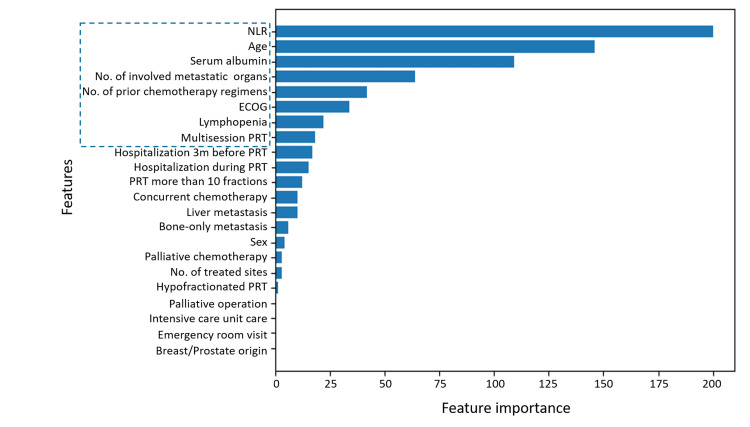
Feature importance with ranking groups Horizontal bars represent the relative importance scores of all input variables, with the top eight most influential features outlined to indicate their grouping as the highest contributors to the model’s predictive performance.

Based on the LightGBM model, test accuracy was highest when the top eight features were used. Increasing the number of features beyond this point resulted in a plateau in accuracy (Figure 1). The top-ranked features in order of importance were NLR, age, albumin, number of involved organs, number of prior chemotherapy regimens, ECOG performance status, lymphopenia, and use of multi-session PRT (Figure 2). Using a minimal variable model (MVM) comprising the top eight features along with the demographic variable sex (nine features in total), predictive performance was comparable to the full variable model (FVM). In fact, recall improved slightly with the MVM (Table 3).

**Table 3:** Comparison of light gradient boosting machine performance between the full variable model and the minimal variable model

	Accuracy	Precision	Recall	Specificity	F1-score
Full variable model	0.725	0.830	0.696	0.771	0.720
Minimal variable model	0.736	0.808	0.750	0.714	0.727

**Figure 3:**
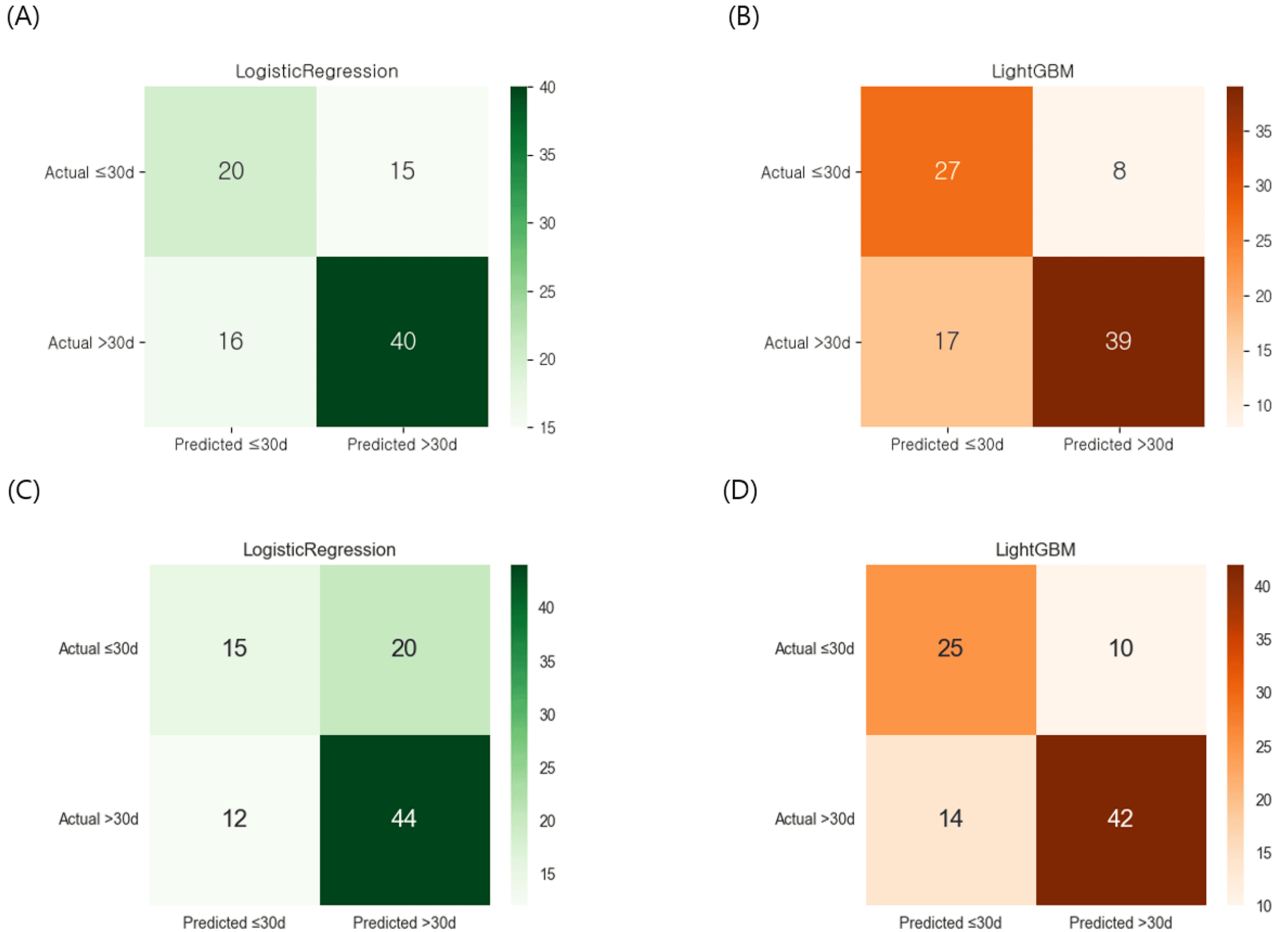
Confusion matrices for logistic regression and LightGBM models using the full variable model and the minimal variable model Panels (A) and (B) display the results of logistic regression and light gradient boosting machine, respectively, trained using the full variable model. Panels (C) and (D) show the results of the same models trained using the minimal variable model, which includes the top eight importance-ranked features and the demographic variable sex. In each matrix, rows represent the actual outcomes (≤30 days vs. >30 days), columns indicate the predicted outcomes, and cell values denote the number of patients in each category.

**Figure 4:**
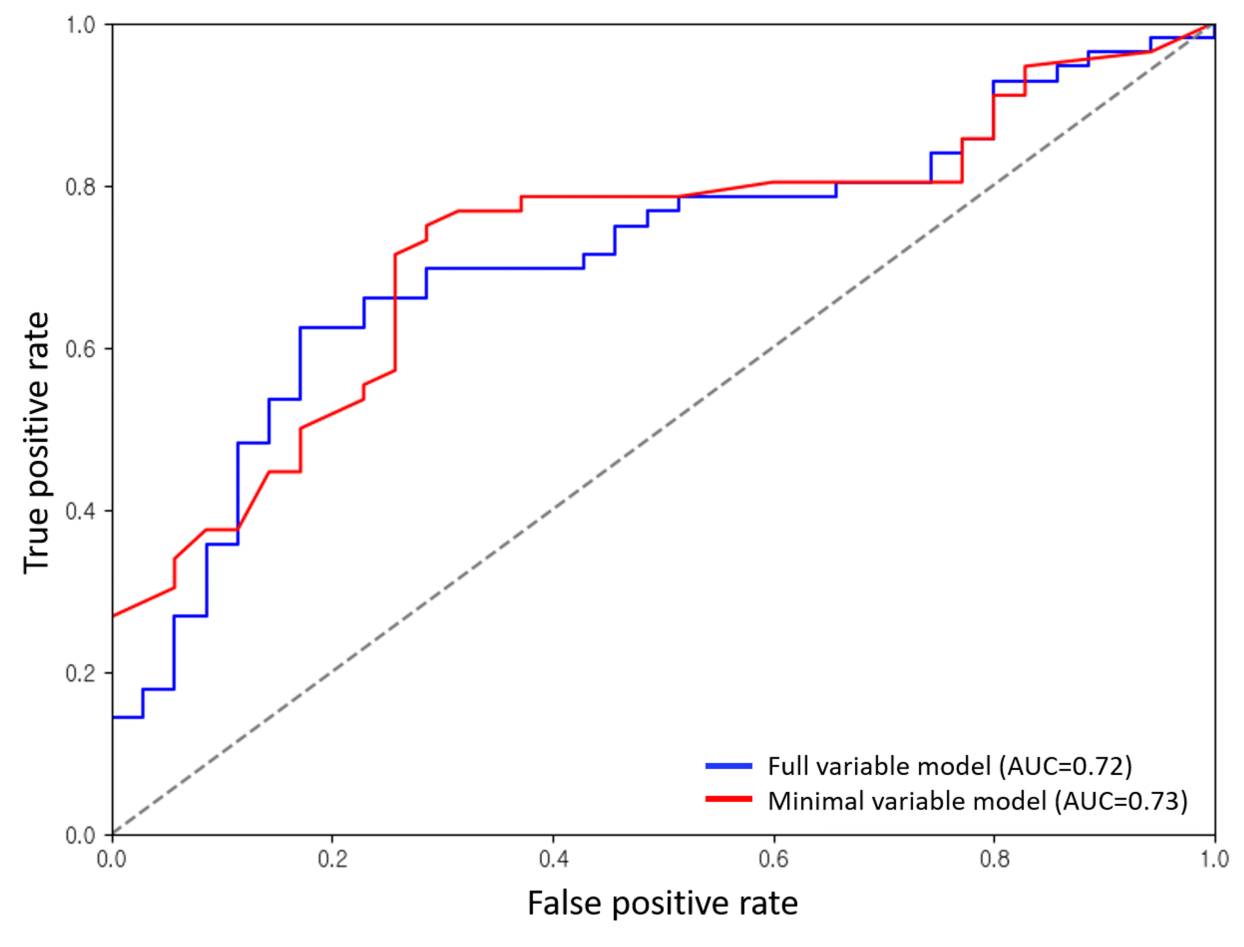
Comparison of AUROC between the full variable model and the minimal variable model Both models demonstrated comparable performance, with minimal difference in discriminative ability. AUROC: Area under the receiver operating characteristic curve.

In the FVM using logistic regression, 20 patients in the <30-day group and 40 patients in the ≥30-day group were correctly classified, while 15 and 16 patients, respectively, were misclassified. In contrast, the LightGBM FVM correctly identified 27 patients in the <30-day group and 39 in the ≥30-day group, indicating better classification accuracy overall. When using the MVM, logistic regression correctly classified 15 and 44 patients in the <30-day and ≥30-day groups, respectively. The LightGBM MVM achieved correct predictions for 25 patients in the <30-day group and 42 in the ≥30-day group, maintaining high accuracy with minimal performance degradation compared to the FVM (Figure 3). The receiver operating characteristic (ROC) curves demonstrated comparable discriminative performance between the two modeling approaches (Figure 4). The MVM achieved an area under the ROC curve (AUC) of 0.73, which was slightly superior to the FVM's AUC of 0.72. The minimal difference in AUC values between the models further supports that the MVM with only the most important features may achieve equivalent or even marginally improved predictive performance compared to the FVM, while offering the advantage of reduced model complexity and enhanced interpretability.

## Discussion

This study aimed to develop a machine learning-based model to predict 30-day mortality in Asian patients with advanced cancer who received PRT. Throughout the model construction process, it was identified that the eight most important variables - peripheral blood NLR, serum albumin, age, number of metastasis-involved organs, number of received chemotherapy regimens, ECOG performance status, lymphopenia, and multisession of PRT - consistently played a crucial role in predicting short-term mortality across different algorithms. The variables significantly impacting model performance were those reflecting the patient’s general condition and tumor burden.

Patients who died within 30 days had significantly higher ECOG grades compared to those who survived beyond 30 days. Among the variables that were statistically significant in Table 1, ECOG, albumin, NLR, and lymphocyte also emerged as top-ranked features in the LightGBM model, demonstrating their importance as predictors of short-term mortality [6,7]. However, some variables such as admission during radiotherapy, despite showing significant differences between groups, had relatively lower feature importance in the model. This suggests that statistical significance alone does not necessarily translate to a high predictive contribution, and that multivariate interactions among variables may play a more critical role in model performance [8].

In this study, we compared MVM using the top eight features plus sex (a demographic variable) with FVM including all available variables. The MVM demonstrated comparable performance to the FVM. As shown in Figure 1, the LightGBM model achieved the highest test accuracy when using eight features, indicating that it is not always necessary to include all variables to build an effective predictive model. Selecting only the most important features can maintain predictive accuracy while reducing the burden of clinical data collection. This approach may enhance the efficiency and consistency of data collection in future multicenter or nationwide big data studies. Indeed, previous studies have reported successful model simplification through feature selection without compromising performance [9,10].

The top eight features identified in our study are clinically meaningful and can explain the risk of short-term mortality in patients with advanced cancer [11-15]. This demonstrates that our model is not a “black-box,” but rather an explainable AI approach. Such explainable AI models can help clinicians trust and interpret predictions, facilitate shared decision-making with patients, and provide valuable guidance for treatment planning [6,9,10,16].

Nevertheless, our study has several limitations. We developed models using LightGBM, XGBoost, random forest, and extra trees algorithms, achieving performance with 0.7 to 0.8. To date, there is no consensus on the optimal algorithm for predicting life expectancy in patients with advanced cancer, and recent studies have reported varying performance across different algorithms, typically highlighting the best-performing one. The performance of our models did not reach the highest levels reported in previous studies, but we believe it is sufficient to assist clinical decision-making. Nonetheless, further improvement is needed for medical AI applications, and future multicenter or nationwide big data studies may enhance predictive performance [17,18].

The relatively small sample size from a single institution is likely the main reason for the modest model performance. A large number of patients were excluded due to missing death dates, reducing the sample size and potentially introducing selection bias, which may have affected the model’s predictive power. To address this, ongoing research is focused on developing models that include patients without confirmed death dates, and collaborative studies with external institutions are underway for external validation. Additionally, laboratory data with high rates of missing values were excluded, limiting our ability to assess their impact on patient outcomes.

Machine learning-based prediction models have not yet been widely implemented in clinical practice, largely due to the lack of standardized reporting and validation procedures, which can lead to biased models and unverified real-world effectiveness [19]. Differences in national healthcare systems and cultural preferences regarding end-of-life care may also affect the importance of certain variables. For example, while emergency room visits have been identified as significant survival predictors in other studies [20], this variable was less important in our model, possibly due to the high accessibility of medical services in South Korea, which may reduce its association with disease severity. Therefore, it is challenging to generalize a single predictive model across all countries, and developing models using large-scale data from countries with similar healthcare systems may help reduce bias.

## Conclusions

In this study, we developed a machine learning-based mortality prediction model to facilitate PRT tailored to individual life expectancy. The top-ranked features identified by the model are factors that have a genuine impact on patient prognosis, highlighting the importance of considering these variables when making treatment decisions for patients referred for PRT. Even without full-variable analysis, the MVM demonstrates potential utility as an auxiliary clinical decision-support tool that could be integrated into clinical workflow to assist clinicians in discussing and determining appropriate care strategies with patients and their families near the end of life. Future prospective and multi-institutional validation studies are warranted to confirm the model’s generalizability and clinical applicability.
